# SCIMP: A Novel Targeted Gene for Postmenopausal Osteoporosis Progression

**DOI:** 10.1111/os.13715

**Published:** 2023-04-14

**Authors:** Xiaolei Sun, Aixian Tian, Peng Li, Jin Zhao, Xin Hou, Xinlong Ma, Xubo Yuan

**Affiliations:** ^1^ Tianjin Key Laboratory of Composite and Functional Materials, School of Materials Science and Engineering Tianjin University Tianjin China; ^2^ Department of Orthopaedics Tianjin Hospital Tianjin China; ^3^ Tianjin Health Information Research Center Tianjin China

**Keywords:** AKT Signaling, Osteoporosis, Postmenopausal Osteoporosis, SCIMP

## Abstract

**Objective:**

The literature suggests that not all postmenopausal women suffer from osteoporosis, and the occurrence of postmenopausal osteoporosis is closely related to the genetic susceptibility of genes in the population and the cellular pathways of related genes. To systematically understand the functions of SCIMP gene for osteoporosis, both in vitro and in vivo experiments were analyzed in depth in this integrated study.

**Methods:**

The significantly differentially expressed genes of postmenopausal osteoporosis (PMOP) patients from GEO database were selected. Meanwhile, the primary target gene was also confirmed in clinically recruited individuals using ELISA method; 50 postmenopausal osteoporosis patients with a T‐score of ‐2.5 were randomly enrolled; postmenopausal women with a T‐score > −2.5 were included in the non‐osteoporotic group (including osteopenia and normal bone mineral density). The associated processes and signaling pathways were deeply investigated with GO and KEGG enrichment analysis. The downstream signaling factors including Erk‐1/2, Akt, and IkB‐related signaling pathways for the potential gene were evaluated using MG‐63 cell line; the MTT, CCK‐8, and flow cytometry assays were performed to exam MG‐63 cell viability, proliferation, as well as apoptosis, respectively, under different treatments.

**Results:**

Based on the differentially expressed gene analysis for GEO database, PMOP patients displayed 845 differentially expressed genes, including 709 down‐regulated and 136 up‐regulated ones. Ten genes including SCIMP were significantly differentially expressed (at least three‐fold difference). SCIMP was the most markedly decreased in PMOP patients’ specimens. Using clinical recruited individuals, the concentration of SCIMP was 96.6 ± 20.8 ng/μL in the PMOP group compared with 168.8 ± 23.5 ng/μL in the control group (*p* < 0.05). At the same time, the osteoclast differentiation signaling pathway was significantly up‐regulated while hedgehogs as well as other signaling pathways were down‐regulated based on the KEGG analysis. The phosphorylation level of Akt was markedly blocked in si‐SCIMP treatment. Up‐regulation of SCIMP increased cell proliferation, inhibited cell apoptosis, and enhanced cell viability in MG‐63 cells, which was markedly rescued by AKT phosphorylation inhibitor. Finally, in vivo experiments also confirmed that the upregulation of SCIMP enhanced the structural parameters of rat trabecular bone and the osteogenic activity of bone tissue.

**Conclusion:**

SCIMP plays a critical role in the pathogenesis of postmenopausal osteoporosis in women. SCIMP influences osteoclasts function through an akt‐dependent molecular pathway, and subsequently influences the equilibrium process of bone metabolism. This provides a new insight into the pathogenesis of postmenopausal osteoporosis as well as the clinical treatment of osteoporosis.

## Graphical abstract

SCIMP as a key regulator for proliferation, apoptosis as well as viability of MG‐63 cells via Akt signaling. (A) The phosphorylation levels of AKT (p‐AKT) compared with levels of total AKT (t‐AKT). (B) The cell proliferation in different treatments. (C) Flow cytometry method was conducted to evaluate cell apoptosis. (D) The MTT assay was performed to analyze cells

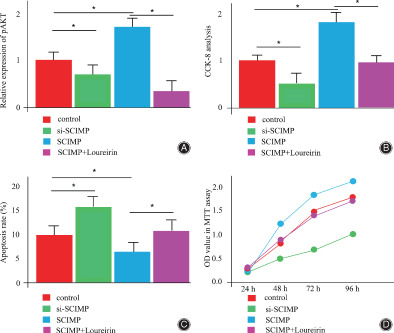

## Introduction

Osteoporosis is a disease with significantly reduced bone mass and microstructural destruction that leads to fragility fractures.^1^, ^2^ According to a survey by the WHO, about 30% of women are affected by osteoporosis during their lifetime.^3^ Although menopause is a close relationship with the occurrence of osteoporosis in women, not all postmenopausal women have osteoporosis.^4^ Postmenopausal osteoporosis precautions include a healthy diet (such as a high‐protein diet), adequate calcium and vitamin d intake, and proper exercise.^5^ Clinical treatment of osteoporosis is preferred by bisphosphonates combined with basic therapy such as calcium and vitamin d supplementation.^6^ Therefore, it is critical to explore the pathogenic mechanism of susceptibility genes in postmenopausal osteoporosis.

There are several risk factors contributing to the pathophysiology of osteoporosis for PMOP patients, including continuously mechanical loading of the skeleton, leading to microdamage, estrogen deficiency, aging, and oxidative stress.^7^, ^8^, ^9^ For postmenopausal osteoporosis (PMOP), it has been suggested that estrogen deficiency is the most likely cause. Additionally, there are also multiple nonheritable and heritable risk factors resulting in the development of osteoporosis and the occurrence of fragility fracture. The nonheritable factors consist of nutrition deficiency, smoking cessation, soft tissue padding, as well as other severe illness.^10^ Even with dramatic improvements over the past decades, the understanding for genetical mechanisms behind osteoporosis is still limited. Currently, a large proportion of genes and polymorphisms have been suggested as potential candidates for the bone mass determination, including transforming growth factor β1 (TGF‐β1), bone morphogenic proteins (BMPs) and Akt signaling pathways.

The Akt signaling, also named as protein kinase B (PKB) pathway, was discovered 25 years ago and has been initiated as the focus of tens of thousands of studies in diverse fields of biology and medicine. At this moment, Akt signaling has been demonstrated to ubiquitously function in nearly every cell, dysfunction of which could result in a variety of human disorders including several types of cancers, developmental defects and overgrowth syndromes, inflammatory and autoimmune disorders, neurological disorders, and bone disorders, etc.^11^ Previously, a study by Chen and his colleagues suggested that a novel epitranscriptomic marker (M6A methyltransferase METTL3) was functional via PI3K‐Akt signaling to stimulate the expression of bone formation‐related genes (such as Runx2 and Osterix), which was closely associated with adipogenesis differentiation and osteogenic differentiation.^12^ In immune cells, the transmembrane adaptor protein (TRAP) family plays a central role in scaffolding signaling proteins and transmitting extracellular information.^13^ SCIMP represents SLP adaptor and C‐terminal Src kinase (CSK)‐interacting membrane protein, which is characterized as the most recently described member of the pTRAP family. The SCIMP has been shown to interact with Src family kinases to regulate MHC class II signaling in B cells and Dectin‐1 signaling in dendritic cells.^14^

Collectively, based on the facts of unclear molecular mechanisms behind the PMOP patients, this study is mainly focused on the following three key points: (i) by GO and KEGG enrichment analysis, examine the associated signaling pathways with the differentially expressed genes for PMOP; (ii) analyze the downstream factors of SCIMP for PMOP formation; (iii) further confirm that SCIMP intervenes in osteoclasts through the Akt pathway to influence the development of PMOP.

## Material and Methods

### Differential Gene Analysis

The information of mRNA sequencing information was obtained from the GEO database (GEO, https://www.ncbi.nlm.nih.gov/geo/), numbering GSE56116, which contained three PMOP patients and three control participants. The gene expression was measured using the Affymetrix Human Genome U95 Version 2 Array platform (http://www.affymetrix.com/products_services/arrays/index.affx) following evaluating with limma package in R language (Version 4.0, Raleigh, US). The absolute value of the log‐transformed differential expression multiple (Log2FC) > 1 and *p*‐value <0.05 were considered as a criterion.

### ELISA Analysis of SCIMP

Fifty PMOP patients (age: 56.3 ± 7.6 years) were randomly selected and recruited in Tianjin hospital from January 2017 to January 2021 as the PMOP group. Fifty female participants without osteoporosis or other bone disorders (age: 57.8 ± 9.3 years) were set up as the control group. All the individuals were confirmed with osteodensitometry by DEXA (Dual Energy X‐ray Absorptiometry) (Discovery w, Hologic). The diagnostic criteria for osteoporosis in this study are based on DEXA, including the T‐score of lumbar spine 1–4 and hip (femoral neck and total hip). Postmenopausal women with T‐score ≤ −2.5 were included in the osteoporosis group. Postmenopausal women with T‐score > −2.5 were included in the non‐osteoporosis group (including osteopenia and normal bone density). Although many postmenopausal women suffer from osteoporosis, there are also many postmenopausal women who only suffer from reduced or even normal bone mass (t value > −2.5). Osteopenia and normal bone density were included in the non‐osteoporotic group in this study. This study was in line with the medical ethics standards and approved by the hospital ethics committee (No. 2017–113). All treatment and testing had the informed consent of patients or their families. The concentrations of SCIMP were determined by ELISA double antibody sandwich method from patients' peripheral blood. The specific operation was carried out in strict accordance with the instructions of the kit (Abcam).

### Functional Enrichment Analysis

The ClusterProfiler package in R language was processed for Gene ontology (GO) analysis (including Biological Process, Molecular Function and Cellular Component) as well as Kyoto Encyclopedia of Genes and Genes (KEGG) pathway enrichment analysis.^15^

### Cell Culture

The MG‐63 cell line was used for the PMOP functional experiments in this study. The cell line (Cobioer) was cultured in MEM/DMEM‐H 10% FBS medium with 1% penicillin/streptomycin. The subcultured cells were used for analysis. The SCIMP or si‐SCIMP vector plasmid was transfected into THP‐1 cell line using Liposome 3000 (Fisher).

For the AKT phosphorylation (p‐AKT) inhibition experiments, MG‐63 cells were treated with Loureirin A (MCE, China) at a recommended concentration of 100 μM.

### Functional Experiments in MG‐63 Cells

The cell viability was measured using MTT assay (Fisher, China) at 24 h, 48 h, 72 h, and 96 h of the culture period. After that, the cells were incubated for another 4 h at 37°C following by 150 mL dimethyl sulfoxide (Sigma) treatment for 10 min. The cells from each group were collected and evaluated for the proliferation assay using CCK‐8 method (Fisher). The absorbance was evaluated at 450 nm using the plate reader purchased from Thermo Fisher Scientific Co. Meanwhile, the cell apoptosis was measured using flow cytometry after Annexin V FITC/PI double staining.

### Western Blotting Analysis

The MG‐63 cells were harvested and washed twice with phosphate‐buffered saline (PBS). The protein concentration of cell lysate was determined using the Pierce BCA protein assay kit (Thermo Fisher Scientific). Thirty milligrams of proteins were loaded on premade 8%–15% SDS polyacrylamide gel for separation and then electrotransferred onto a nitrocellulose membrane. The blots were incubated in PBS buffer with 5% defat milk and 0.02% Teween‐20 at room temperature for 1 h before primary antibody incubation overnight at 4°C. All the antibodies were obtained from Jackson Immunoresearch Laboratory, United States. The antibodies of phospho‐Akt (p‐AkT), phospho‐IkB (p‐IkB), phospho‐Erk (p‐Erk), total Akt (t‐AkT), total IkB (t‐IkB) as well as ErK (t‐Erk) were utilized with a 1:1000 dilution, respectively. The SuperSignal West Pico Chemiluminescent Substrate (Thermo Fisher Scientific) was used for visualization of immunoreactive proteins.

### Animal Experiments

Healthy 6‐month‐old female Sprague–Dawley rats (body weight, 260 ± 12 g) were provided by the Experimental Animal Center of Tianjin Hospital. The experimental animals received humane care, and the study protocols conformed to the guidelines of Tianjin Hospital Ethics Committee (No.20200132). The animals were housed in an air‐conditioned environment (21 ± 3°C) with a 12‐h light/dark cycle and were allowed free access to food pellets and water throughout the experiment. Thirty rats were randomly divided into three groups (10/group), and two groups were subjected to bilateral removal of the ovaries (OVX). Ovariectomy was performed under anesthesia with chloral hydrate (400 mg/kg, IP) by removing the bilateral ovaries together with their capsules and part of the oviduct through a dorsal approach. Surgical incisions were all sutured with a 5–0 synthetic absorbable suture. The rats in the sham group were subjected to the same incision to remove the same volume of adipose tissue around the bilateral ovaries sutured without removal of the ovaries. After 60 days of surgery, the plasmid vector or SCIMP plasmid vector was transfected into OVX group. The transfection complex was prepared by mixing 5 nmol SCIMP plasmid vector, 40 μL Entranster‐in vivo transfection reagent (Engreen), and 40 μL of 10% glucose for 15 minutes at room temperature according to the manufacturer's instructions. Then, medullary cavity injections were performed with SCIMP plasmid vector or plasmid vector and Entranster‐in vivo complex at 3 weeks after OVX.

### Micro‐CT Analysis

After DEXA measurements, the trabecular bone microarchitecture of the distal right femoral metaphysis was measured using a microtomography scanner (SkyScan 1076, Kontizh, Belgium) with a slice thickness of 21 μm and the voxel resolution of 22 μm^3^.

The volume of interest (VOI) was selected as a region 25–125 slices away from the distal femur growth plate. The 3D images were obtained for visualization and display. Bone morphometric parameters, including bone volume over total volume (BV/TV), trabecula number (Tb.N), and trabecula thickness (Tb.Th), were obtained by analyzing the VOI. Bone morphometry can accurately reflect the microstructural changes of the bone tissue. (BV/TV), (Tb.N), and (Tb.Th) can observe the total volume and quantity of the trabecular bone and the thickness of the bone.

### Histological Analysis

Five right femurs from each group were fixed with 75% ethanol and collected for decalcification sections and immunohistochemical staining. A light microscope (TE2000U, Nikon) was used to view the decalcification sections and HE stain. The parameters were analyzed by Image‐Pro Plus 6.0 software (Media Cybernetics).

### Statistics

The experimental results were repeated three times independently and were tested by statistical methods. The SPSS 22.0 statistical software (IBM Corp. Released 2013. IBM SPSS Statistics for Windows, Version 22.0: IBM Corp.) was developed for data analysis. The continuous variables were tested for normal distribution and the student *t*‐test in SAS 9.4 was used to analyze the data, with *p*‐value <0.05 was considered as statistically significant.

## Results

### The Investigations of the Differentially Expressed mRNAs

**Fig. 1:**
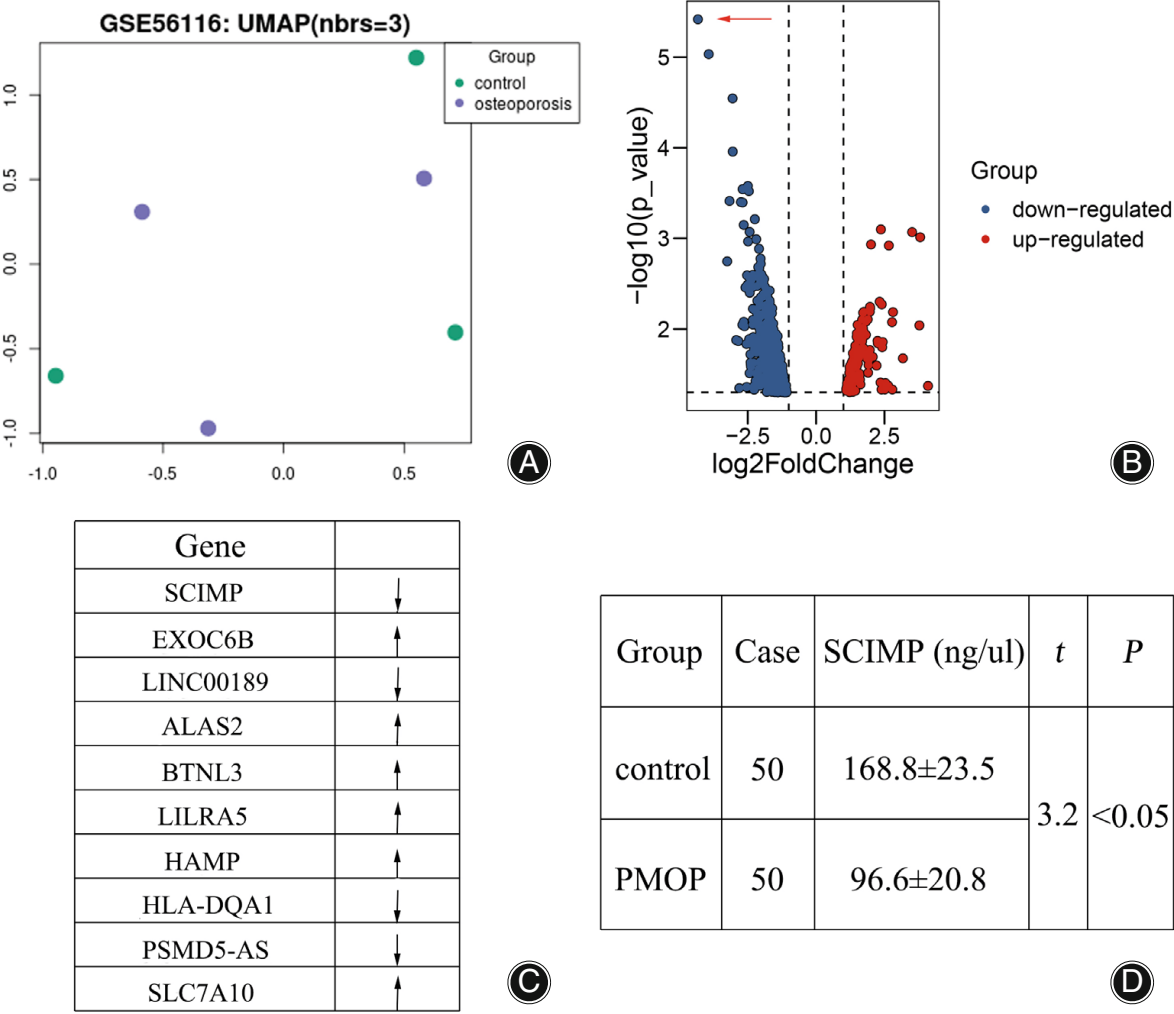
Investigation of differentially expressed genes. (A) The UMAP algorithm analysis of PMOP patients and control participants. (B) The volcano map of differentially expressed genes. The horizontal axis is the multiple of differential expression (Log2FC), the vertical axis is ‐log10 (FDR), while the blue dot represents down‐regulated genes and the red dot represents up‐regulated genes, respectively. The red arrow indicates the most significantly down‐regulated gene (SCIMP). (C) The primary differentially expressed genes for PMOP patients compared with control specimen. (D) The ELISA investigation of SCIMP expression level using clinical recruited individuals

This study first compared the overall expression pattern between PMOP patient and sibling control by UMAP algorithm analysis. As shown from Figure 1A, the two groups of participants did not demonstrate striking expression models. Based on the differentially expressed gene analysis, PMOP patients displayed 845 differentially expressed genes, including 709 down‐regulated and 136 up‐regulated ones (Fig. 1B). Among these, *SCIMP, EXOC6B, LINC00189, ALAS2, BTNL3, LILRA5, HAMP, HLA‐DQA1, PSMD5‐AS*, and *SLC7A10* were significantly differentially expressed (at least a three‐fold difference, Fig. 1C). SCIMP was the most markedly decreased in PMOP patients (shown as red arrow in Fig. 1B). Based on the ELISA results, the concentration of SCIMP was 96.6 ± 20.8 ng/uL in the PMOP group compare with 168.8 ± 23.5 ng/uL in the control group (*p* < 0.05) (Fig. 1D).

### GO and KEGG Enrichment Analysis

**Fig. 2:**
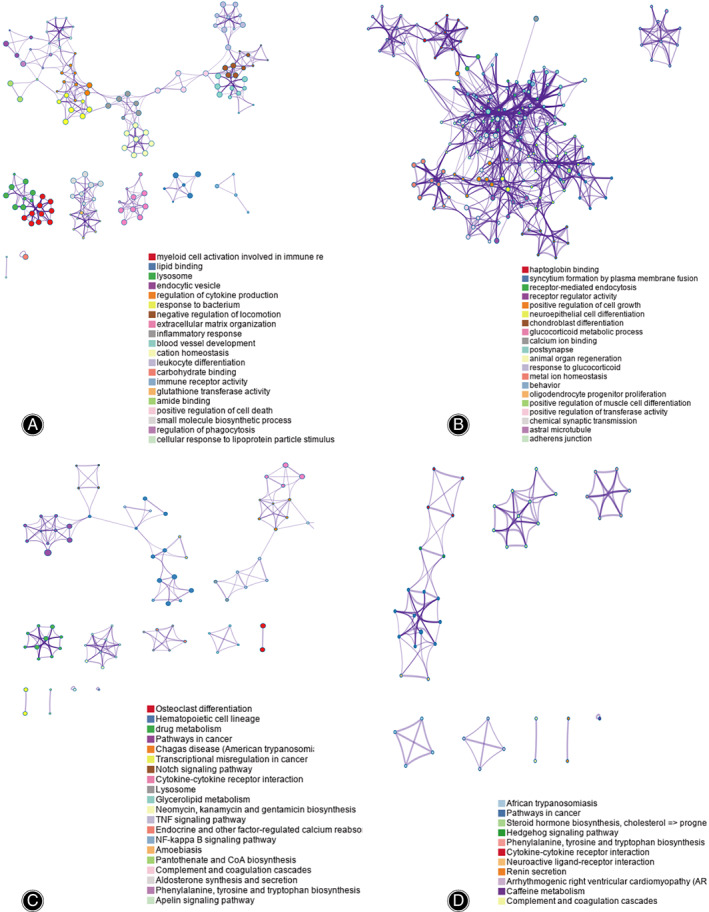
GO and KEGG enrichment analysis for PMOP patients. (A) The primary up‐regulated GO term enrichment results with the largest number of genes. In the figure, the horizontal axis indicates the number of enriched genes, while the vertical axis indicates the name of each GO term, respectively. (B) The top down‐regulated GO term enrichment results. (C) The enrichment results of the up‐regulated KEGG pathways with the largest number of genes. The horizontal axis in the figure represents the number of genes enriched, and the vertical axis represents the name of each KEGG pathway, respectively. (D) The enrichment results of the down‐regulated KEGG pathways with the largest number of genes

By performing GO and KEGG enrichment analysis on these 845 differentially expressed genes, it could be seen that these potential genes were significantly enriched in GO terms such as cell activation involved in immune response, lipid binding, etc. (up‐regulation) (Fig. 2A) and plasma membrane fusion, receptor mediated endocytosis etc. (down‐regulation) (Fig. 2B). At the same time, the osteoclast differentiation signaling pathway was significantly up‐regulated (Fig. 2C) while hedgehog as well as other signaling pathways were down‐regulated (Fig. 2D) based on the KEGG analysis.

### Effects of SCIMP in MG‐63 Cells

**Fig. 3:**
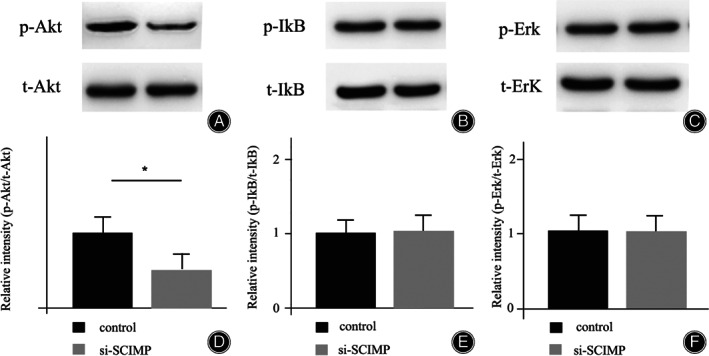
SCIMP is required for Akt signaling responses in MG‐63 cells. (A‐C) The western blotting for the evaluation of phospho‐Akt (p‐AkT), phospho‐IkB (p‐IkB), and phospho‐Erk (p‐Erk), respectively. D‐F is the corresponding relative chemiluminescence. The t‐AkT, t‐IkB, and t‐Erk represent the expression of total Akt, IkB as well as ErK

Previously, it has been reported that SCIMP manipulated several signaling modules including Erk‐1/2, Akt, and IkB as a phosphorylation accelerator in macrophage.^16^ To testify whether this is same for osteoporosis formation, the phosphorylation levels of Erk, Akt, and IkB in MG‐63 cells were measured. As the activity of SCIMP was inhibited by si‐SCIMP transfection, there was no apparent change for the phosphorylation levels of Erk and IkB (Figs 3B,C). Yet again, the phosphorylation level of Akt was markedly attenuated in si‐SCIMP treatment (Fig. 3A).

### SCIMP as a Key Regulator for Proliferation, Apoptosis as Well as Viability of MG‐63 Cells

**Fig. 4:**
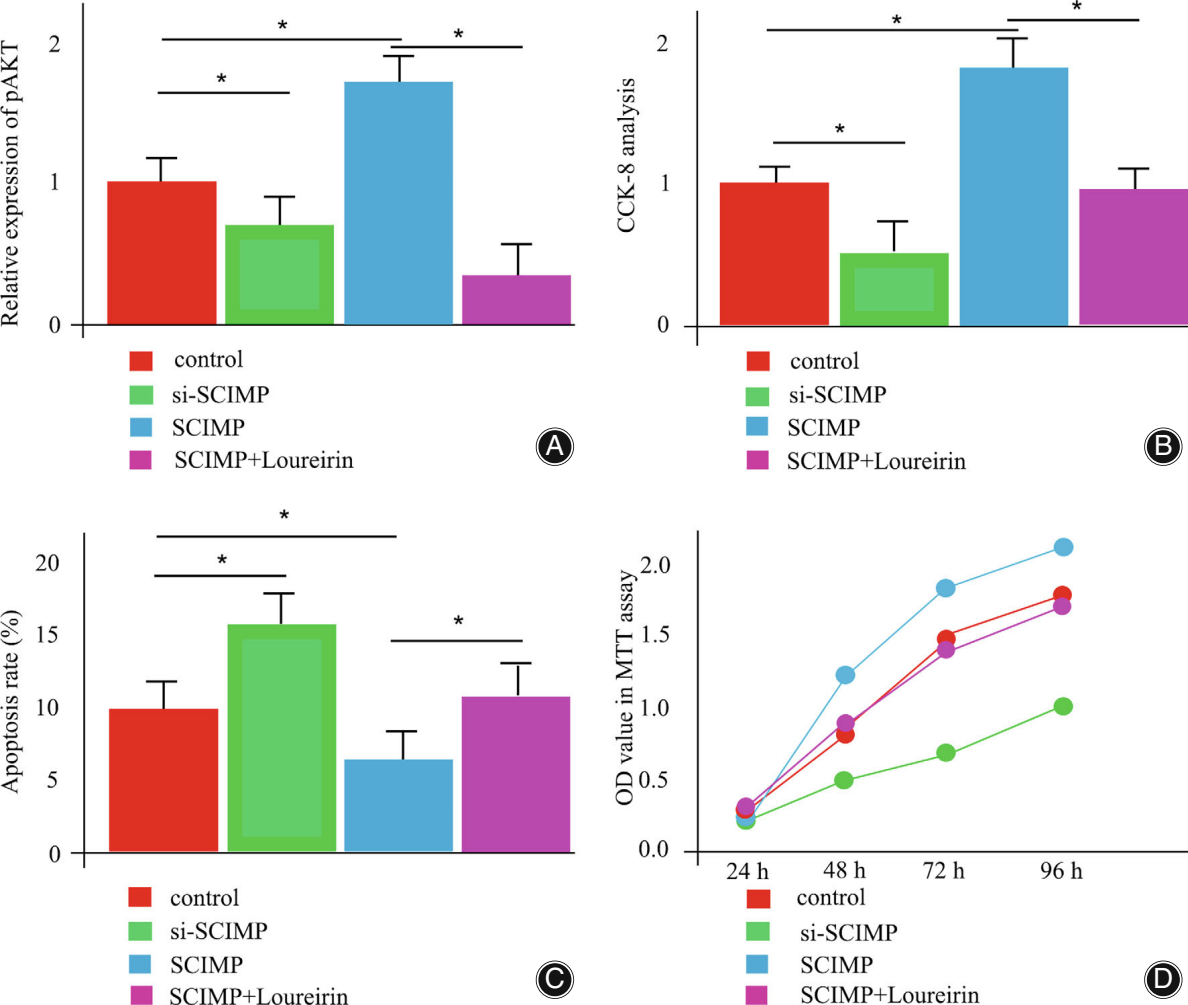
SCIMP is responsible for proliferation, apoptosis, as well as viability of MG‐63 cells. (A) The western blotting was used to measure the phosphorylation levels of AKT (p‐AKT) compared with levels of total AKT (t‐AKT). (B) The CCK‐8 assay was used to exam cell proliferation in different treatments. (C) Flow cytometry method was conducted to evaluate cell apoptosis. (D) The MTT assay was performed to analyze cell, respectively

The MG‐63 cells were transfected with si‐SCIMP or SCIMP plasmid vector to introduce decreased or increased expression respectively. Compared with control, SCIMP overexpression was able to stimulate AKT phosphorylation (p‐AKT), which was compensated by AKT phosphorylation inhibitor, Loureirin A (Fig. 4A). At the same time, the introduction of si‐SCIMP could significantly reduce cell proliferation while SCIMP overexpression was able to enhance the cell proliferation, which was blocked by co‐treatment of Loureirin A (Fig. 4B). Moreover, downregulation of SCIMP stimulated and upregulation of SCIMP attenuated cell apoptosis, which was rescued by Loureirin A (Fig. 4C). The MTT assay further supported that SCIMP transfection markedly stimulated cell viability, which was inhibited by si‐SCIMP or Loureirin A treatment (Fig. 4D).

### SCIMP Overexpression Abrogated OVX‐Induced Trabecula Decline and Akt Protein Levels Reduction in OVX Rats

**Fig. 5:**
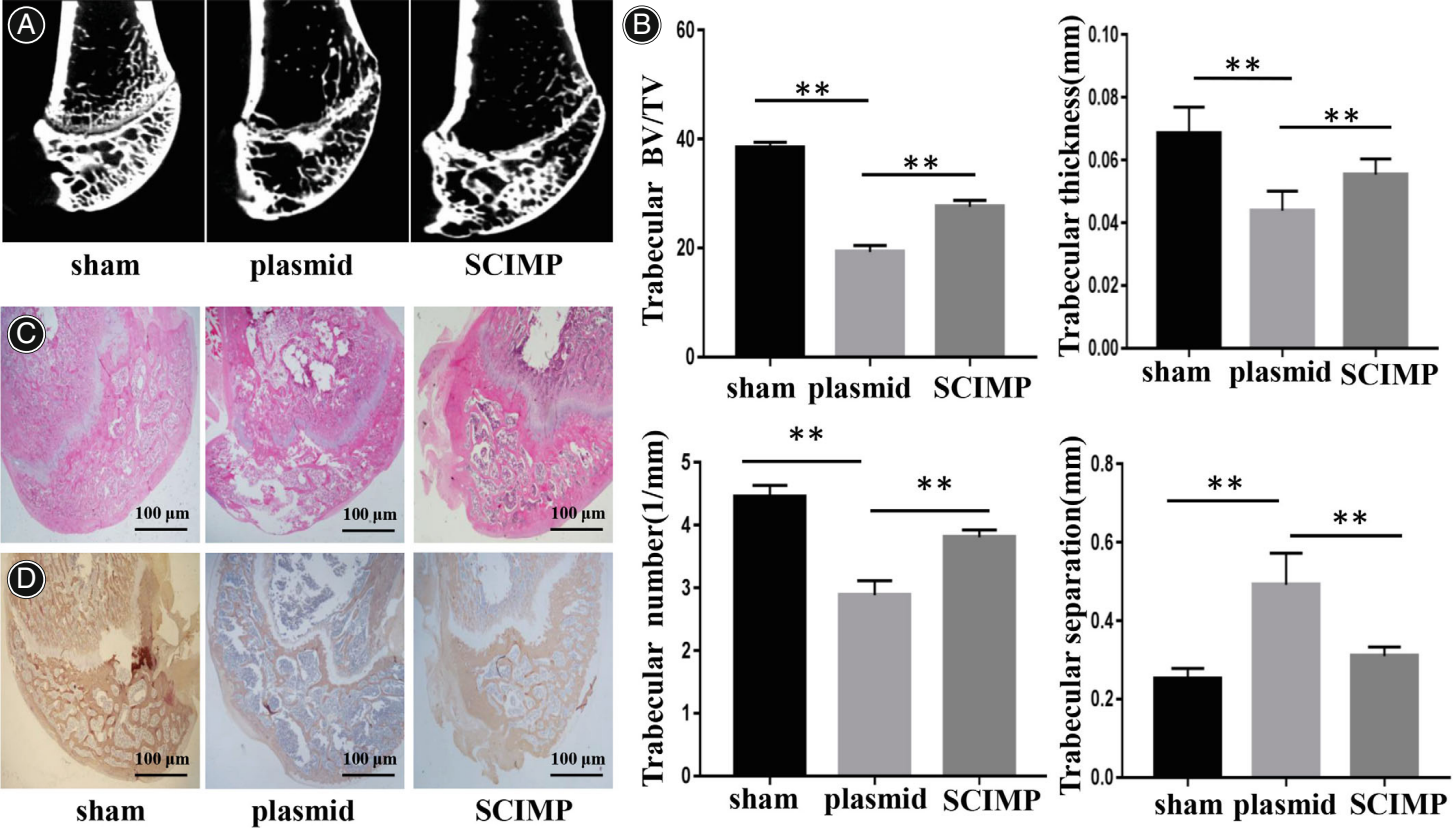
(A) Representative Micro‐CT images of distal femoral tissues in VOX rat groups. Compared with sham group, there were looser trabecular bones in SCIMP group in vivo. (B) Quantification of trabecular bone structure within the ROI in Micro‐CT images calculated trabecular BV/TV, trabecular thickness, trabecular number, and trabecular separation. (C and D) Representative H&E and immunohistochemical staining of trabeculae at the distal femur. Scale bar = 100 μm. **p < 0.05

The SCIMP plasmid vector was injected in vivo into the rat femoral canal. Effects of SCIMP on trabecular microstructure two‐dimensional and three‐dimensional reconstructed images from micro‐CT scanning showed microarchitecture of trabecular bone in distal femoral metaphysis of all rats (Fig. 5A). Compared with sham group, there were looser trabecular bones in OVX group. In the SCIM plasmid vector group, the BV/TV, trabecular number, and trabecular thickness in the distal femur were considerably increased compared to values in the plasmid vector group, while trabecular separation was decreased (Fig. 5B). H&E staining showed consistent results with micro‐CT (Fig. 5C). Compared with sham group, decreased osteogenic function was observed in the OVX group. The levels of Akt protein were higher in the SCIMP plasmid vector group than the plasmid vector group (Fig. 5D).

## Discussion

It has been reported that the incidence of postmenopausal osteoporosis is closely related to the genetic susceptibility of the population.^17^ The results of this study found that SCIMP plays a key role in the pathogenesis of postmenopausal osteoporosis. SCIMP inhibits the activity of osteoclasts through the akt‐dependent molecular pathways, which subsequently affect postmenopausal skeletal metabolic processes.^18^ In vivo animal experiments further confirmed that SCIMP plays an active role in the reconstruction of bone mass and trabecular microstructure. To this end, we sought to explore the molecular mechanisms underlying the disease.

### Detection of PMOP Differentially Expressed Genes and Their Related Signal Pathways

It is worth noting that postmenopausal women suffer from a high rate of osteoporosis. Moreover, postmenopausal women are normally at a risk of morbidity and mortality for both breast cancer and PMOP. What is the underlined connection for the two mayhems? Estrogen is an important issue that cannot be ignored.^19^ Indeed, based the study from a large German population, PMOP patients administrated with hormone replacement therapy also demonstrated a lower risk of colorectal cancer.^20^ This was closely associated with the expression level change of estrogen. Direct evidence was found in a prospective cohort study by Limsui et al. who claimed that postmenopausal hormone therapy was inversely connected to distinct molecularly defined colorectal cancer subtypes and this relied on the differential effects from estrogen.^21^ Estrogen is a well‐known potent steroid with pleiotropic effects, with both nuclear and non‐nuclear functions. The response to estrogen depends on the proper interactions between a membrane associated estrogen receptor (ER) and TRAP family members.^22^ Here, based on the gene differentially expression analysis, SCIMP was a central candidate for the process of PMOP (Figs. 1B‐D). As a most recently identified member of the TRAP family, SCIMP might be a primary plasma factor responding to extracellular estrogen. Akt signaling has been suggested as a critical downstream factor for estrogen and the signaling cassette has become more complex and interdependent with acquired endocrine resistance. AZD5363, a novel pan‐AKT kinase catalytic antagonist has been reported to influence estrogen receptor functions in breast cancer based on an *in vivo* study.^23^ In this study, we could show that the functions of SCIMP for proliferation, apoptosis and viability of osteoblast was Akt signaling dependent (Fig. 4), suggesting a potential molecular mechanism for PMOP formation. Nevertheless, we emphasized the downstream signaling pathway without estrogen involvement, which might be a meaningful direction for the next step.

### Downstream Factors and Cellular Functions of SCIMP for PMOP Formation

This study verified the sequestration of smart MG‐63 cells differentiation by the Akt pathway using MTT assay and western blotting analysis at the cellular level. Previously, the report by Luo and his colleagues implied that deletion of SCIMP could result in decreased phosphorylation of Erk, Akt, and IkB in macrophages.^16^ In their study, SCIMP‐mediated tyrosine phosphorylation was shown to be a crucial response to a variety of pathogens of the innate immune system and SCIMP was a universal adaptor for proximal Toll‐like receptor. Furthermore, SCIMP was also suggested as an autonomous on/off switch by scaffolding both positive and negative regulators of inflammatory signaling responses, which subsequently selectively activate and deactivate the adaptive immune system.^24^, ^25^ These characteristics provide the lymphocytes with broader range of opinions to modulate signaling outputs.^26^ In this study, we did not detect measurable phosphorylation for Erk and IkB (Fig. 3). There are two possibilities for this. First of all, Erk and IkB signaling pathways are not as important as Akt signaling pathway for PMOP processes. Secondly, the SCIMP is not ubiquitously functional as in lymphocytes and the partnering with membrane associated estrogen receptor imparts specificity for osteoclast. We preferred the second explanation since there are several reports supporting the connection between osteoporosis and Erk as well as IkB signaling pathways.^27^, ^28^, ^29^ On the other hand, it is difficult to rule out these two signaling cassettes completely due to their widespread roles.

### Validation of Up‐Regulation of SCIMP Expression in PMOP Formation In Vivo

In some previous studies, SCIMP has been found to play a central role in trabecular bone reconstruction in rats.^30^ But can SCIMP also ameliorate bone mineral density loss or trabecular structural destruction in osteoporotic trabecular reconstruction? In our study, it was confirmed that the effective upregulation of SCIMP was specific in the formation of PMOP in rats. The injection of SCIMP plasmid significantly improved the BV/TV, trabecular number, and trabecular thickness in the distal femur in PMOP rats. Moreover, transfection with SCIMP plasmid effectively eliminated type II collagen content in distal femur bone tissue in vivo. Compared with the existing literature,^16^ our results further confirm that the clinical application value of gene site‐specific targeting is obvious in the treatment of osteoporosis and in predicting the onset of osteoporosis. Furthermore, upregulation of SCIMP in vivo abolished the osteoporotic histopathological changes and trabecular decay of the rat femur. The results confirm that up‐regulation of SCIMP in vivo effectively prevents the progression of postmenopausal osteoporosis.

### Strengths and Limitation

Based on the existing clinical methods for osteoporosis treatment, this study focused on the pathogenesis genes and action pathways of postmenopausal osteoporosis, by screening possible susceptibility genes and verifying them in in vivo and in vitro experiments. It provides a new direction for the research ideas and potential clinical treatment of osteoporosis. Even with the systematical analysis here, several limitations should also be considered. Initially, the differentially expressed genes profile was based on the online database (no. GSE56116), which contained three PMOP patients and three control participants. Since the recruited individuals were restricted, we also utilized 50 PMOP patients to verify the potential target genes. However, compared with the large population of PMOP, 50 PMOP participants might not be enough and there should be a cohort study with a large patient sample size as well as multi‐center investigation in the future. Meanwhile, we focused on Erk‐1/2, Akt, and IkB as the key downstream factors for SCIMP based on previous publications. However, we could not rule out other critical signaling pathways for SCIMP functions in PMOP processing.

## Conclusion

All in all, this study highlighted SCIMP as a central factor for PMOP development and formation, which manipulated osteoclast cellular functions through a Akt‐dependent molecular mechanism. All the work here provided a novel insight for future postmenopausal osteoporosis study.

## Authorship Declaration

Xiaolei Sun, Peng Tian, and Jin Zhao performed experiments. Peng Li and Xin Hou contributed to data interpretation. Xiaolei Sun, Xinlong Ma, and Xubo Yuan designed experiments. All authors have read and approved the final submitted manuscript. The authors declare no competing financial and nonfinancial interests.

## Funding Information

This work was supported by grants from the National Nature Science Foundation of China (No. 31600769; No. 81871777).

## Disclosure

The authors declare no conflict of interest.
